# United states of amnesia: rescuing memory loss from diverse conditions

**DOI:** 10.1242/dmm.035055

**Published:** 2018-05-18

**Authors:** Clara Ortega-de San Luis, Tomás J. Ryan

**Affiliations:** 1 School of Biochemistry and Immunology, Trinity Biomedical Sciences Institute, and Trinity College Institute of Neuroscience, Trinity College Dublin, Dublin 2, Ireland; 2 Florey Institute of Neuroscience and Mental Health, Melbourne Brain Centre, University of Melbourne, Parkville, VIC 3052, Australia

## Abstract

Amnesia – the loss of memory function – is often the earliest and most persistent symptom of dementia. It occurs as a consequence of a variety of diseases and injuries. These include neurodegenerative, neurological or immune disorders, drug abuse, stroke or head injuries. It has both troubled and fascinated humanity. Philosophers, scientists, physicians and anatomists have all pursued an understanding of how we learn and memorise, and why we forget. In the last few years, the development of memory engram labelling technology has greatly impacted how we can experimentally study memory and its disorders in animals. Here, we present a concise discussion of what we have learned about amnesia through the manipulation of engrams, and how we may use this knowledge to inform novel treatments of amnesia.

## Abstract (precis)

**Summary:** We examine cases of memory loss caused by diverse interventions, diseases and injuries, and summarize how engram investigation and manipulation may help us understand and potentially treat amnesia.

## Introduction

Amnesia refers to a deficit of memory due to a specific cause. It is a disorder that arises as a consequence of more than 15 different types of diseases and injuries that affect the brain, such as neurodegenerative and neurological diseases, vascular disorders and traumatic lesions (Markowitsch and Staniloiu, 2012). It is often the earliest and most persistent symptom of dementia (Wells, 1979). Amnesia has a huge clinical significance – its effects on the daily life of patients who suffer from it can be enormous. As a result, there are many efforts towards developing successful treatments. Currently, therapeutic interventions are limited by the lack of understanding of how memory functions in both health and disease.

Memory is the ability to store information of past experiences in the brain. Knowledge learnt by animals alters their brain and modulates how the brain then regulates future behaviour. Understanding memory, and its mechanisms, is a central goal of modern neuroscience. In 1904, Richard Semon postulated that experiences provoke long-lasting changes in specific neurons that result in an enduring memory trace – an engram of the acquired information. Reactivation of these engram cells will result in the recall of that particular memory (Semon, 1904). To understand the mechanisms of engram formation, we have primarily relied on indirect methodological approaches, for example, by studying amnesia. The general approach is to interfere with a brain region, physiological process or gene that we hypothesize is important for memory, and then look for experimental amnesia in a given behavioural paradigm (McGaugh, 2000). Recently, we have begun to make progress in our understanding of both memory and amnesia through the development of memory engram technology.

**Fig. 1.**
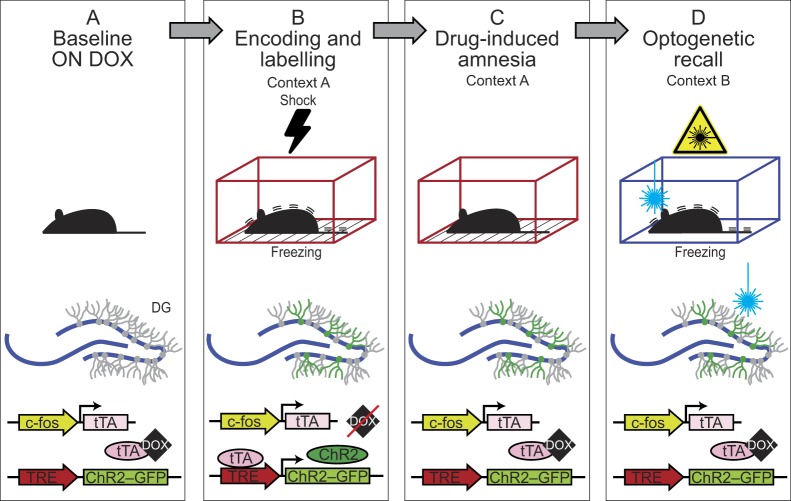
**Engram labelling technology and memory retrieval in retrograde amnesia.** (A) The promoter of the immediate early gene (IEG) drives expression of tTA in an activity-dependent manner. Doxycycline (DOX), which is delivered through the animal's diet, prevents tTA from binding to the TRE element of the channelrhodopsin (ChR2) transgene in hippocampal dentate gyrus (DG) neurons. (B) Shock delivery, which causes fear, in context A subsequently elicits a freezing response specifically to context A. In the absence of DOX, DG neurons that are active during the encoding of that fear memory express ChR2. Injection of the drug anisomycin after the encoding induces retrograde amnesia. (C) Amnesic animals are unable to elicit a behavioural (freezing) response using natural cues. (D) Optogenetic activation of engram neurons induces the recall of a distributed and context-specific fear response in amnesic animals.

Memory engram technology is based on the combination of transgenic, optogenetic, behavioural and electrophysiological approaches. Developed originally by Tonegawa and colleagues, the technology integrates optogenetics and immediate early gene (IEG) labelling to drive the expression of a transgene in cells that specifically respond to an experience (Boyden et al., 2005; Reijmers et al., 2007; Tonegawa et al., 2015a,b). In its first demonstration, a promoter of the IEG *c-fos* was used to drive the expression of channelrhodopsin (ChR2), a light-responsive ion channel, in hippocampal dentate gyrus neurons that were activated by a target contextual experience (Fig. 1). Temporal control is allowed by the tetracycline-controlled transactivator (tTA)-tetracycline response element (TRE) system, inducible by the removal of the antibiotic doxycycline so that it only labels the neurons that are responding to the controlled contextual experience. This approach demonstrated that direct activation of engram neurons for contextual memories associated with fear/threat is sufficient (Liu et al., 2012; Ramirez et al., 2013), as well as necessary (Denny et al., 2014; Tanaka et al., 2014; Trouche et al., 2016), to recall this specific episodic memory.

## Amnesia

From the clinical point of view, amnesia is described as a multifaceted disorder with a frequently poor prognosis (Markowitsch and Staniloiu, 2012). Anterograde amnesia refers to the inability to acquire and retain new information, whereas retrograde amnesia affects the recall of past or recently learned memories. Recent memories are more vulnerable to amnesia than older ones, and this is known as Ribot's law of regression (Ribot, 1881). Amnesia appears as a consequence of diverse clinical disorders, such as Alzheimer's and Parkinson's disease (AD and PD, respectively), depression, and head trauma, among many others. Therefore, animal models for those disorders frequently develop memory deficits (Table 1).

**Table 1.**
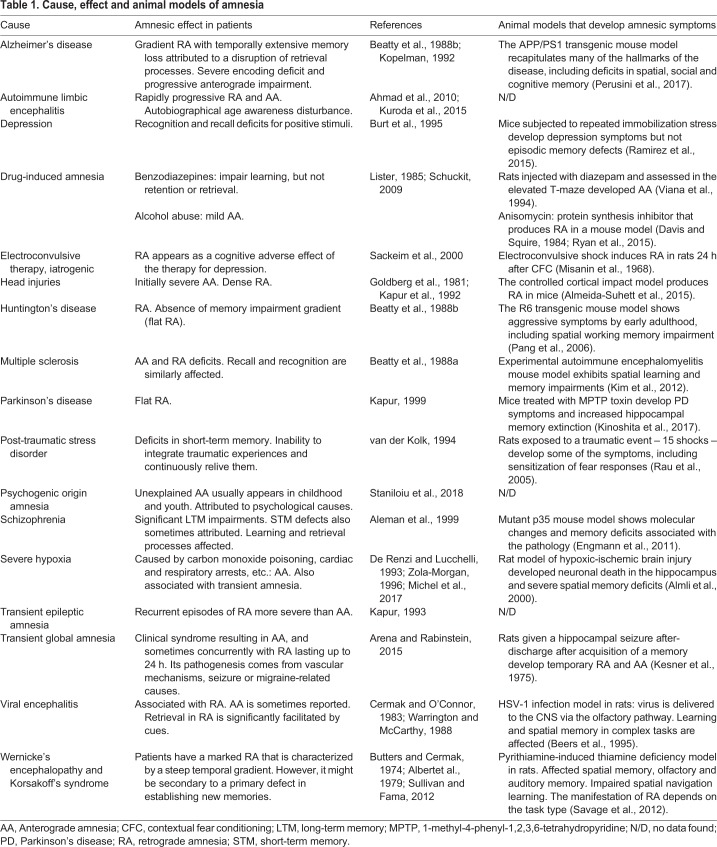
**Cause, effect and animal models of amnesia**

Can the combination of memory engram labelling technology and disease models help us to understand the neuropathology of amnesia? In a model of drug-induced amnesia, mice administered with a protein synthesis inhibitor after a fear-inducing training session develop retrograde amnesia for that fear memory (McGaugh, 2000). Surprisingly, using the engram labelling technology, optogenetic activation of neurons in these mice elicited a context-specific fear response, indicating that the memory was still there (Fig. 1) (Ryan et al., 2015). This approach opens the possibility that the information is not completely lost in retrograde amnesia, but it is just inaccessible.

The same methodological approach was subsequently applied to models of early AD – the major neurodegenerative disease that affects memory storage (Roy et al., 2016; Perusini et al., 2017). AD is associated with the deposition of amyloid-β peptide in extracellular plaques and with the aggregation of the microtubule-associated protein tau in neurofibrillary tangles inside neurons (Braak and Braak, 1991). As a consequence of these aggregates, synapses are compromised, and there is selective neuronal death and a decrease in specific neurotransmitters (reviewed in Masters et al., 2015). The APP/PS1 mouse model recapitulates many of the hallmarks of human AD, including deficits in spatial, social and cognitive memory (Gong et al., 2004; Lalonde et al., 2005), but some strategies have successfully improved cognition in these models. Environmental enrichment was shown to be beneficial by stimulation of synaptic activity (Jankowsky et al., 2005; Lazarov et al., 2005; Fischer et al., 2007). Photonic stimulation of the visual cortex by chronic application of light in frequencies of 40 Hz improved contextual and fear memory, and significantly reduced amyloid-β plaque deposition (Iaccarino et al., 2016). Although these studies show that certain activities and interventions can ameliorate the deterioration of memory, they do not show whether the engram itself survives amnesia. In the APP/PS1 mouse model, short-term memories (minutes to hours) are intact, whereas long-term memories (a day or more) are compromised, indicating a consolidation deficit as a cause of the amnesia (Kilgore et al., 2010; Ryan et al., 2015). The fact that this kind of amnesia is retrograde (because the initial short-term memory is observed) indicates that the engram might still be present in the brain. Using the engram tagging approach in this model, animals with amnesia due to early-stage AD were able to remember a contextual memory through optogenetic stimulation of the labelled engram neurons. This proved that, firstly, the memory is maintained and, secondly, the cells responsible for encoding the original memory are not properly reactivated in early-stage AD models (Roy et al., 2016; Perusini et al., 2017). Furthermore, engram technology has provided insights into the role of memory loss in depression. Based on human studies, it has been hypothesized that depression may be due in part to a loss of access to positive memories (Dalgleish and Werner-Seidler, 2014). Engram technology has provided strong experimental evidence in favour of this idea. When positive or pleasurable memory engrams were labelled in the mouse hippocampus prior to the induction of depression, subsequent optogenetic stimulation of these engram cells ameliorated depressive behaviour, and chronic stimulation seemed to induce new plasticity in those cells that restored natural access to the engram and normal behaviour thereafter (Ramirez et al., 2015).

## Treatment of amnesia

The idea that the information survives in the context of the pathology is changing the paradigm of amnesia and instigating the search for therapeutic strategies to make seemingly lost memories obtainable again, rather than simply preventing the memory loss in the first place. Such therapeutics would have wide-ranging utility, since amnesia is a common symptom of many different brain disorders (Table 1). The first objective will be to identify which kinds of retrograde (and perhaps in some cases, anterograde) amnesia are due to retrieval deficits. The subsequent step will be to find ways to restore access to those engrams in a sustainable manner. Animal studies are best placed to achieve both these initial aims before the strategies can be translated into human clinical cases. Importantly, any treatment or intervention designed to reverse amnesia, whether chronic or acute, needs to also be tested in control wild-type animals. This is crucial to account for general cognitive effects (e.g. arousal, attention, emotional response, etc.) that might affect behavioural performance independently of any improvement of memory engram function.

However, such approaches to ameliorate or reverse amnesia need to be complemented with continuing efforts to address the underlying cause of the disorder. This is especially the case for chronic forms of amnesia, as the memory will become inaccessible again if the problem is still present, such as in neurodegenerative disorders. Since amnesia is not the primary cause of these diseases, efforts put into finding therapies that palliate the memory deficits should be tied to therapies designed to stop the overall progress of the amnesia-causing disease in question.

What achievements in treating amnesia should be expected in the short and long term? Therapies based on optogenetic stimulation are very invasive, and this is a major obstacle to their translation to the clinic. The light required to optogenetically stimulate labelled neurons needs to be delivered through optic fibres, and gene therapy is required to make cells susceptible to light-mediated activation. This limitation might be overcome in the future by the development of the next-generation optogenetic implantable devices (Zhao and Hubin, 2017; Rudmann et al., 2018; Shoffstall et al., 2018) and by optimization of gene delivery (Dobson, 2006; Wang et al., 2017). Examples of non-invasive alternatives to target and activate engrams are transcranial direct-current stimulation (tDCS) and transcranial magnetic stimulation (TMS). tCDS applies weak electrical currents with electrodes that either hyperpolarize or depolarize the neurons to modify brain function. TMS achieves the same effect by generating a magnetic field inside a coiled wire that in turn generates an electrical field at the intracranial level. Although safer and much less invasive, the benefits of tDCS and TMS on cognitive function in AD are acute and not maintained in the long term (Freitas et al., 2011), probably because of their lack of specificity in targeting neurons. Most crucially, unlike researchers, physicians are not generally present at the time of memory engram formation in the patient's brain, and so are not in the privileged observational position to label human engrams in clinical cases, even if safe and appropriate technology was available. However, given the progress in the past 10 years, there is every reason to be optimistic about future possibilities of overcoming this caveat.

As memory engram technology has become available as a new tool, the memory research field has advanced in its understanding of memory storage, consolidation and retrieval processes. Combining these approaches with disease models associated with amnesia will help us better understand the pathology on a neurobiological level, and this would certainly be followed by better management and therapeutic treatment of patients affected by memory loss.
